# Dynamic Perceived HIV Risk and Sexual Behaviors Among Young Women Enrolled in a PrEP Trial in Kenya: A Qualitative Study

**DOI:** 10.3389/frph.2021.637869

**Published:** 2021-08-12

**Authors:** Kenneth Ngure, Nicholas Thuo, Vallery Ogello, Catherine Kiptinness, Kevin Kamolloh, Bridget Frances O'Rourke Burns, Nelly R. Mugo, Elizabeth A. Bukusi, Lindsey Garrison, Jared M. Baeten, Jessica E. Haberer

**Affiliations:** ^1^Department of Community Health, Jomo Kenyatta University of Agriculture and Technology, Nairobi, Kenya; ^2^Department of Global Health, University of Washington, Seattle, WA, United States; ^3^Center of Clinical Research, Kenya Medical Research Institute, Nairobi, Kenya; ^4^Center for Microbiology Research, Kenya Medical Research Institute, Nairobi, Kenya; ^5^Department of Urban Studies and Planning, Massachusetts Institute of Technology, Cambridge, MA, United States; ^6^Center for Global Health, Massachusetts General Hospital, Boston, MA, United States; ^7^Department of Obstetrics and Gynecology, University of Washington, Seattle, WA, United States; ^8^Center for Global Health, Massachusetts General Hospital, Boston, MA, United States; ^9^Gilead, Foster City, CA, United States; ^10^Department of Epidemiology, University of Washington, Seattle, WA, United States; ^11^Department of Medicine, University of Washington, Seattle, WA, United States; ^12^Department of Medicine, Harvard Medical School, Boston, MA, United States

**Keywords:** pre-exposure prophylaxis, HIV, risk perceptions, sexual behaviors, women, Kenya

## Abstract

**Background:** In Kenya and elsewhere in sub-Saharan Africa, young women are disproportionately affected by the HIV epidemic compared to young men. The extent to which young women's self-perceptions about risk of HIV acquisition influence their sexual behaviors and use of HIV prevention methods remains unclear. We therefore conducted a qualitative study to explore these issues among young women enrolled in a pre-exposure prophylaxis (PrEP) trial.

**Methods:** From January 2017 to January 2020, we conducted serial semi-structured in-depth interviews 50 purposively selected young women (18–24 years old) who were participating in the MPYA (Monitoring PrEP for Young Adult women) study—a randomized controlled trial in Thika and Kisumu, Kenya, assessing the impact of SMS reminders on PrEP adherence. Interviews were conducted at three time points (~1 week, 3, and 12 months after initiating PrEP). We used an inductive, content analytic approach to identify key themes related to risk perceptions, sexual behavior, and use of HIV prevention tools.

**Results:** Around the time of enrollment, most of the 50 women interviewed reported being at high risk of HIV because of their own sexual behaviors, such as inconsistent condom use, multiple sexual partners, and transactional sex. Additionally, high risk perception was based on the behavior of their partners, such as refusing to use condoms and being unsure of their partner's HIV status. Young women's perceived risk of HIV acquisition was a key motivator for PrEP initiation and continuation. During PrEP use, participants reported feeling protected and at less risk compared to peers who were not taking PrEP. Some reported no longer using condoms because they were confident that PrEP provided enough protection. Over time, many young women reported reducing risky sexual behaviors because of the regular counseling and HIV testing they received as part of their PrEP services. This lowered risk perception was in most cases accompanied by discontinuation of PrEP.

**Conclusions:** HIV risk perception among young women in Kenya was dynamic and influenced their use of PrEP and condoms over time, suggesting an often-deliberate approach to HIV prevention and sexual health.

## Background

Adolescent girls and young women (aged 15–24) in many parts of sub-Saharan Africa (SSA) are at a much higher risk of HIV acquisition compared to young men (1, 2). Factors that increase this risk include inability to negotiate condom use, intimate partner violence, social expectations such as early marriages, and biological factors such as presence of sexually transmitted infections (STIs) (2, 3). Social cognitive theories, such as the Expanded Health Belief Model and Theory of Reasoned Action, postulate that individuals who perceive risk of acquiring a disease will be more likely to engage in risk reducing sexual behaviors (4–6). Therefore, interventions that target HIV risk perception could result in improvements of healthy sexual behaviors.

Although objective risk scoring tools have been developed, validated, and used to identify populations at risk (7, 8), they do not necessarily generalize across different environments or perform well-over time (9). Additionally, risk scores do not always align to risk perceptions—yet it is the perceptions that have been shown to influence sexual behavior and potentially adherence to interventions, including HIV pre-exposure prophylaxis (PrEP). For example, in a recent study among Kenyan and Ugandan HIV serodiscordant couples, high-risk sexual behavior aligned with self-reported PrEP adherence, suggesting participants may have been motivated by risk perception (10). Another analysis from the same study found alignment of sexual behavior and perceived risk, although risk perception did not influence electronically measured PrEP adherence (11). These findings evince the complexity in understanding perceptions and associated behaviors.

Daily oral PrEP with tenofovir disoproxil fumarate and emtricitabine (TDF-FTC) is an efficacious HIV prevention tool when used consistently and is currently being scaled up globally (12). The World Health Organization (WHO) recommends PrEP for persons at “substantial” risk of HIV—guidance that has been adopted and included in country specific guidelines including in Kenya (13, 14). However, challenges in adherence have been reported among young women in sub-Saharan Africa both in clinical trials and recent implementation science projects (15–20). These studies have reported that despite high PrEP awareness and being at high risk of HIV PrEP uptake and continuation has been poor even when PrEP has been provided onsite. Some of the reasons for poor PrEP uptake and continuation include low risk perception (21, 22). In the Monitoring PrEP for Young Adult women (MPYA) study, PrEP adherence was generally low with no difference between arms (23). There is therefore a need to understand the multiple factors that drive HIV risk behavior among AGYW at risk of HIV infection which could benefit not only the uptake and continuation of biomedical agents but also other HIV prevention methods as well (24).

Most HIV risk and sexual behavior studies date has been quantitative and have used a cross-sectional study design (11, 24). For example, in VOICE and FEM-PrEP studies where <30% of the women had detectable tenofovir, assessment risk perception was based on a single quantitative question which in itself was inadequate to measure risk (15, 16). We therefore conducted a prospective qualitative study to explore in-depth whether young women recognized their HIV risk and how these perceptions of HIV risk and sexual behaviors evolve over time among young women enrolled in a PrEP trial.

## Methods

### Study Design and Setting

From January 2017 to January 2020, we conducted serial in-depth interviews with young women enrolled in the MPYA study. The MPYA study was a randomized trial conducted in two Kenyan sites (Thika and Kisumu) (23), Thika is an urban center in Kiambu County, about 40 km outside of Nairobi, with a large surrounding peri-urban and rural population and estimated HIV prevalence of 1.1%; the Kisumu site is located in Western Kenya in Kisumu County and serves both an urban and a large fishing community. The estimated HIV prevalence among adults aged 15–64 is 1.1 and 17.5% in Kiambu County and Kisumu County, respectively (25). The MPYA trial assessed the effects of SMS reminders on PrEP adherence over 2 years of participant follow-up. PrEP adherence was measured with a real-time adherence monitor and on-going risk assessments were collected by weekly SMS surveys in all participants. We used a multipronged community-based recruitment strategy for young women at risk of HIV, which included recruiting women from farming communities, informal settlements, organizations providing primary health care, HIV clinics (for young HIV uninfected women in serodiscordant relationships). The eligibility criteria for the young women to be enrolled into the MPYA trial included age 18–24 and being at high risk of HIV at enrolment as per the VOICE risk score (i.e., a score > 5) (7). Specifically, the VOICE risk score is based on age, marriage, or living with a husband or primary sexual partner, provision of financial or material support by partner, the partner having other sexual partners, and alcohol use. Within the context of the MPYA trial, the young women received regular counseling, HIV testing, risk assessment, and provision of condoms and clinical care free-of-charge.

### Sampling

For the serial interviews, we used stratified purposeful sampling to ensure we selected young women from each of the two participating sites, ages <21 and >21 years of age, and the intervention vs. control arms (26). To ensure spread over the whole MPYA cohort, we invited every 7th participant within each category. Interviews were conducted around the time of enrolment (within 1 week of PrEP initiation), 3, and 12 months. Participants were invited to attend all three interviews; the Kisumu site added other participants from the same cohort at 3 and 12 months if the initial participant was not available.

### Data Collection

We used a semi-structured interview guide to ask participants about their understanding of PrEP adherence, including barriers and facilitators, as well as their understanding of HIV risk behavior, risk perceptions, motivation for HIV prevention, and how these factors relate to sexual behavior (see Supplementary Material). Concepts in cognitive psychology were used to guide questions around risk perception (27–30). Interviews were conducted face-to-face by experienced social scientists, led by authors NBT and VO, in a quiet, private space using the women's preferred language (Dholuo, Kiswahili, or English) at a time mutually convenient for the participants and study staff. All interviews were audio-recorded, transcribed, and translated into English when necessary. The interviews lasted an average of 43 min.

### Data Analysis

We used a combination of inductive and deductive approaches informed by the Expanded Health Belief Model to identify key themes (31). First, transcripts were reviewed through an iterative process as the data came in weekly to identify emerging themes. We then developed a coding scheme through a second review of a randomly selected subset of one-third of interview transcripts. Operational definitions were then developed for the codes to create a codebook, which was used to code the data (32). Four analysts (KN, KO, NBT, and VO) read through all the transcripts, and NBT and VO coded sections of the transcripts that appeared to address HIV risk perceptions and sexual behavior, including condom use, using an agreed upon codebook. KN, KC, and SV reviewed the coded transcripts for code agreement. HIV risk perceptions and sexual behavior data was then compared across the three serial time points. We used the qualitative data management software, Dedoose (dedoose.com), to code the data, which was then be repeatedly sorted and re-reviewed to identify a broader set of concepts.

### Ethics

The study was approved by the Kenya Medical Research Institute Scientific and Ethical Research Unit (KEMRI-SERU) and the University of Washington Human Subjects Division. All participants provided written informed consents.

## Results

### Participant Characteristics

A total of 50 young women participated in the in-depth interviews at the first serial time-point; the number of participants at each time point is shown in Table 1. Participants had a median age of 21 years (interquartile range [IQR] 20–22), median years of education of 12 (IQR 10–13), and median VOICE risk score of 7 (IQR 6–7); 54% (*N* = 27) reported more than one sexual partner (Table 2). The serial interviews describe how the young women's risk perceptions and sexual behaviors evolved during study follow-up under the following themes guided by the Expanded Health Belief Model: (1) HIV risk perception, sexual behavior at enrolment, and perception of HIV/AIDS, (2) high risk perception as a motivator for PrEP initiation and continuation, (3) risk perception and sexual behaviors after initiating PrEP, and (4) reduced risk perception leading to PrEP discontinuation.

**Table 1:** Number of interviews by time period.

**Site**	**Enrolment**	**Month 3**	**Month 12**
Thika	25	18	17
Kisumu	25	25^*^	25^*^
Total	50	43	42

* *Kisumu site replaced four participants during follow-up interviews though their data was not included in the serial analysis*.

**Table 2:** Baseline demographic characteristics of study participants (*n* = 50).

**Characteristics**	***N* (%) or Median (IQR)**
Age (years)	21 (20, 22)
Education (years)	12 (10, 13)
VOICE risk score	7 (6, 7)
Sex acts in prior month	3 (2, 6)
>1 total current sexual partner	27 (54)
Any condomless sex in past month	25 (50)
Partner provides support	21 (42)
Sexual relationship power^*^	2.6 (2.4, 2.8)
Sexual intimate partner violence	9 (18)
Possible depression^**^	24 (48)
Alcohol use	8 (16)
Travel to study site >1 h	15 (30)

*IQR, interquartile range; N, number participants*.

* *The Sexual Relationship Power Scale (25) reflects the median of 15 items with Likert responses; scores range from 1 to 4 with higher scores indicating less power within the relationship*.

** *Depression was assessed by the PHQ-2 (26) (a response of “yes” to either question is considered as possible depression)*.

### HIV Risk Perception, Sexual Behavior, and Perception of HIV/AIDS at Enrolment

In the first serial interviews, most young women reported being aware of their HIV risk, stating “*we know these things*.” Many perceived themselves as being at high risk of HIV acquisition as a result of their own sexual behaviors, such as having multiple partners, new partners at parties and under the influence alcohol, and sex in exchange for money or gifts. This high-risk perception motivated some of the women to go for HIV testing frequently to confirm their HIV status. Only four women during the first serial interviews specifically reported that they did not understand their risk very well, stating “*You never know*.”

“*Oooh yes, aaah in the past I have been at risk a lot and you know I am a person who likes going for raves (parties) and you know when you are drunk you do a lot of things and maybe I have sex with someone so you get worried in the morning because you don't know this person, his HIV status. You don't know what he does, how many people he sleeps with (engaging in sex)*” (*IDI#25, 20 years old, single with no steady partner—serial IDI 1*).

“*I thought I was at high risk and could barely wait for 1 month without going to be tested… I did not like sex, I feared it, and even if someone gave me a date, I had to lie to him because I felt that I was at risk and could not even save myself. Even those Trust (condom brand), I did not trust them*” (*IDI#1, 22 years old, single with steady partner—serial IDI 1*).

High risk perception at the first serial interviews was also reportedly driven by the high-risk behavior of the young women's partners, who they did not trust. Others reported being at high risk based on common community beliefs about the high-risk behavior of men. Being single or with unsteady partners was also reported to be a risk factor, especially because the young women did not live with them and therefore were not sure of their behaviors when they were not together. Many of the young women additionally reported that they were unaware of their partners HIV status and that their partners often refused to be tested. The young women further reported that that their partners often refused to use condoms especially during transactional sexual relationships (N.B., participants referred to these partners as “sponsors”). Most participants in our study were well aware that HIV risk could be a potential consequence of their own and their partners' sexual behaviors.

“*Considering that I am not yet married, I may have a partner who maybe goes out there and has many relationships and can be stressing me*” (*IDI#19, 21 years old, single with no steady partner-serial IDI 1*).

“*It's because I have many partners and they don't like using condoms and I don't know their status because they refuse to get tested*” (*IDI#11, 23 years old, single with no steady partner—serial IDI 1*).

“…* when you meet someone for the first day, you know this issue of sponsor? So you know that this guy's needs this and that in order to give you this and you reach a point and tell him to put on a condom and he says he does not use that as there are people who do not use them. So, you have to take care of yourself and be one step ahead*” (*IDI#18, 22 years old, single with no steady partner—serial IDI 1*).

Overall participants reported that AIDS was a serious disease with many equating HIV with death, especially in cases where people do not adhere to ART. However, some participants reported that ART use would only delay the inevitable AIDS-related death.

“*I: What does HIV/AIDS mean to you as an individual?*

*R: It's a killer disease to me*.

*I: Can you tell me why you say that it's a killer disease?*

*R: When you don't take drugs and eat well, you will die. Definitely (laughter) you have to die*” (*IDI #34, 22-year-old single with no steady partner—serial IDI 1*).

### High Risk Perception as a Motivator for PrEP Initiation and Continuation

The participants' perceived high risk of HIV acquisition was a key motivator PrEP initiation; they wanted PrEP to protect them from HIV infection. The high-risk sexual relationships included those with partners they did not trust and sex with multiple partners and in transactional sexual relationships where the young women reported the greatest challenge in negotiating condom use. Additionally, the young women reported that PrEP would put them at lower risk compared to their peers who were not using PrEP. They also reported that PrEP would offer additional protection if the condom broke.

“*I wanted to take PrEP because am always at risk… You meet different people from different backgrounds and you don't know each other. You only meet for business. I need his cash; he needs my service. So, after service he gives me money and he leaves. I don't know where he comes from. I don't want to know, so long as am done with him. So, when I learnt about PrEP, I said at least it can save me. It can reduce the risk. It can reduce the risk of HIV because at least when I take my drugs, I can be assured that I will not get it*” (*IDI #27, 21 years old, single with no steady partner—serial IDI 1*).

“*I decided to use PrEP because I have many sexual partners and I do not know all their status. I only know the status of two. The rest I do not know their status. That's why I saw that if I come for this PrEP, it is going to help me*” (*IDI#37, 23-year-old, single with steady partner—serial IDI 1*).

“*Anyone who is not taking PrEP is at a higher risk of getting HIV. I am not at risk because I take PrEP. Condoms can burst and that leaves them unprotected still*” (*IDI# 39, 21 years old, single with steady partner— serial IDI 2*).

During the first serial interviews, participants reported that they would use PrEP until they gained trust of their sexual partners or when their relationships stabilized, for example through marriage. They added that if they established that their partners were HIV uninfected (there was a general suspicion that their partners were living with HIV) or became monogamous, they would feel at reduced risk and hence discontinue their PrEP use.

“…*until I am sure the boyfriend is mine or I get married. You know, since now everyone is on his/her own. It is better to stay protected. Yes, when he is safe and also gets tested and I know that everyone is negative*” (*ID#9, 19 years old, single with steady partner—serial IDI 1*).

“*Like for me, if I was married, I wouldn't personally come for PrEP very much because maybe the trust you have for your partner or spouse, the kind of trust is different from the one that you have while you are dealing with other people*” (*ID#20, 18 year old, single with no steady partner—serial IDI 1*).

Individual participants HIV risk perceptions was reported as a key driver for most to initiate PrEP and they added that they would use PrEP as long as they were at risk of HIV acquisition.

### Risk Perception and Sexual Behavior After Initiating PrEP

During the second and third serial interviews, women reported feeling protected and at reduced risk because they were using PrEP. They also reported that PrEP reduced their worry associated with getting HIV. Some women reported low risk throughout the 12 months follow-up, mainly related to their consistent PrEP use. Similar to the time of the first serial interviews women reported at subsequent serial interviews to being at less HIV risk compared to their peers who were not taking PrEP.

“*My stress has reduced since I no longer worry about being infected with HIV. I am taking PrEP now unlike in the past that I was worried about getting HIV after having sex*” (*ID#46, 18 year old, single with no steady partner—serial IDI 2*).

“…*my risk of getting HIV is very low because of this drug PrEP … What makes me think that am at less risk of getting HIV is because of this drug PrEP…*.” (*IDI#37 23-year-old, single with steady partner—serial IDI 2*).

“*Using PrEP has been good because there are some of my friends that we were invited with to come and join the study, but they declined. They thought that they would be used for experiments. There are some who contracted HIV. I thank God that I made the decision to join the study and take PrEP. I am safe and healthy. I have not been infected [remorsefully]. I would be taking ART now if it were not for PrEP. They are sick and do not want to go for care because they feel ashamed. They regret failing to take PrEP because they now have to take ARV for life. I am safe now as compared to the past when I was at a higher risk of getting HIV*” (*ID#39, 21 years old, single with steady partner—serial IDI 2*).

More than half of the participants reported a reduction in condom use during second and third serial interviews. This change in behavior was mainly based on increased trust in PrEP, which made them feel safe even when their partners refused to use condoms. Those in transactional relationships reported that they were now able to engage in condomless sex because they felt protected by PrEP and could therefore obtain more money. Others reported reduced condom use based on reduced HIV risk, reflecting better trust of their partners and becoming aware of partners status (i.e., testing HIV negative as couple, or changing their partners to those they considered lower risk partners). A few maintained their non-use of condoms throughout the follow-up period, stating “*I have never used condom*.”

“*Before I started taking PrEP I was using condoms and after I started taking PrEP I stopped using condoms. I saw that I was okay because I was on a family planning method, I could not get pregnant but I could get HIV. So when I found out that I can also prevent HIV I thought that why not*” (*ID#35, 20 years old, single with steady partner— serial IDI 3*).

“*For now, am not using anything but sometimes I use condoms and sometimes I don't but I am worried because I am not near my partner so am worried about his behaviors*” (*ID#21, 23 years old, single with steady partner—serial IDI 3*).

“*As I have told you my boyfriend was someone who didn't want to use condoms, And that's how I started taking PrEP so when I started taking PrEP and saw that I was consistently taking it, I didn't even bother him with the use of condoms, I just accepted his decision*” (*ID#21, 19 years old, single with steady partner—serial IDI 3*).

Those who indicated continuing with condom use reported using condoms with their casual partners, for pregnancy and STI prevention, and as a back-up in case condoms broke.

“*We just use them because I cannot allow him to go bare [without a condom] because I'm not using any family planning method so I can get pregnant*” (*ID#31, 22-year-old single with no steady partner—serial IDI 2*).

“*We would use condoms when I am not in my safe days only*” (*ID#21, 23 years old, single with steady partner—serial IDI 3*).

“*We can't say that condoms are 100% maybe it might burst, and you don't even know his status*” (*ID#36, 19 years old, single with steady partner—serial IDI 3*).

### Reduced Risk Perception Leading to PrEP Discontinuation

Most participants in the second and third serial interviews reported reducing their risky sexual behavior, such as becoming monogamous or increasing condom use, while others reported that they had become married, which they felt lowered their HIV risk. These risk reduction decisions were based on the regular HIV testing and the counseling they received in the study. The counseling sessions included a risk assessment that participants said motivated them to adopt less risky behaviors. Other participants could not distinguish between PrEP the drug and PrEP services (i.e., the counseling and the HIV testing provided alongside the PrEP) and hence attributed their change in behavior to initiation of PrEP.

“*Okay before I started using PrEP, I think I had many partners and then I was like after being with a partner I was stressed and scared and I just wanted to go to the VCT (voluntary counseling and testing)… And after getting into the study and I knew about PrEP … I can now bargain for safe sex. I can bargain for condoms, yeah and if someone does not want the condoms then I am done with him. We have nothing else to talk about*” (*ID#27, 21 years old, single with no steady partner—serial IDI 3*).

“*You know when I started (joined the study), I had like four men, but I reduced the number to one. Anyone who wants to protect himself will protect himself because even me I am protecting myself as an individual. When we came, we got tested and we found out that we were HIV negative. You'll say I tested HIV negative and the way I have been irresponsible? Then God loves me. Why can't I change? If I decide to change, you say no to those people that are not important to you and they are just after sex, such people you can let go of them … but when I found that I was safe I thought that let me just have one and continue living my life*” (*ID#49, 23 years old, single with no steady partner—serial IDI 3*).

This reduced risk reduction made some discontinue PrEP use, either permanently or for periods of time. They did not feel a need for ongoing HIV protection. That said, one young woman reported being at increased HIV risk since she stopped using PrEP due to PrEP fatigue. Additionally, another participant who was in a HIV serodiscordant relationship reported being in a continuous risk and therefore would not consider discontinuing PrEP.

“*I have not been taking PrEP, reason being my risk is low… I no longer have a partner, so I don't see the reason to take (laughs)*” (*ID#10, 23-year-old, single with steady partner [at enrolment]—serial IDI 2*).

“*I have never taken them [PrEP] on a daily basis. I just take them when I know I will be at risk. Sometimes us women we know these things …, so you just take them before because I was told you don't have to take them every day if you are not at risk. Just take them seven days before and a couple of days after but if you are not at risk at all you don't have to but if you want to you can just take them*” (*ID#25, 20 years single, with no stable partner—serial IDI 3*).

“*I was not a good person so when I became good, I thought that I should stop PrEP, but PrEP itself was good*” (*ID#35, 20 years old, single with a steady partner—serial IDI 3*).

“*Yes, I want to continue with it and also my husband is infected, and he can get tired of taking his drugs and he might do that in secret. So I may get tested 1 day and I find that I'm infected, so I can ask myself how I got infected yet my partner is on drugs but in real sense he stopped taking the drugs*” (*ID#40, 24-year-old, single with steady partner—serial IDI 3*).

## Discussion

**Figure 1:**
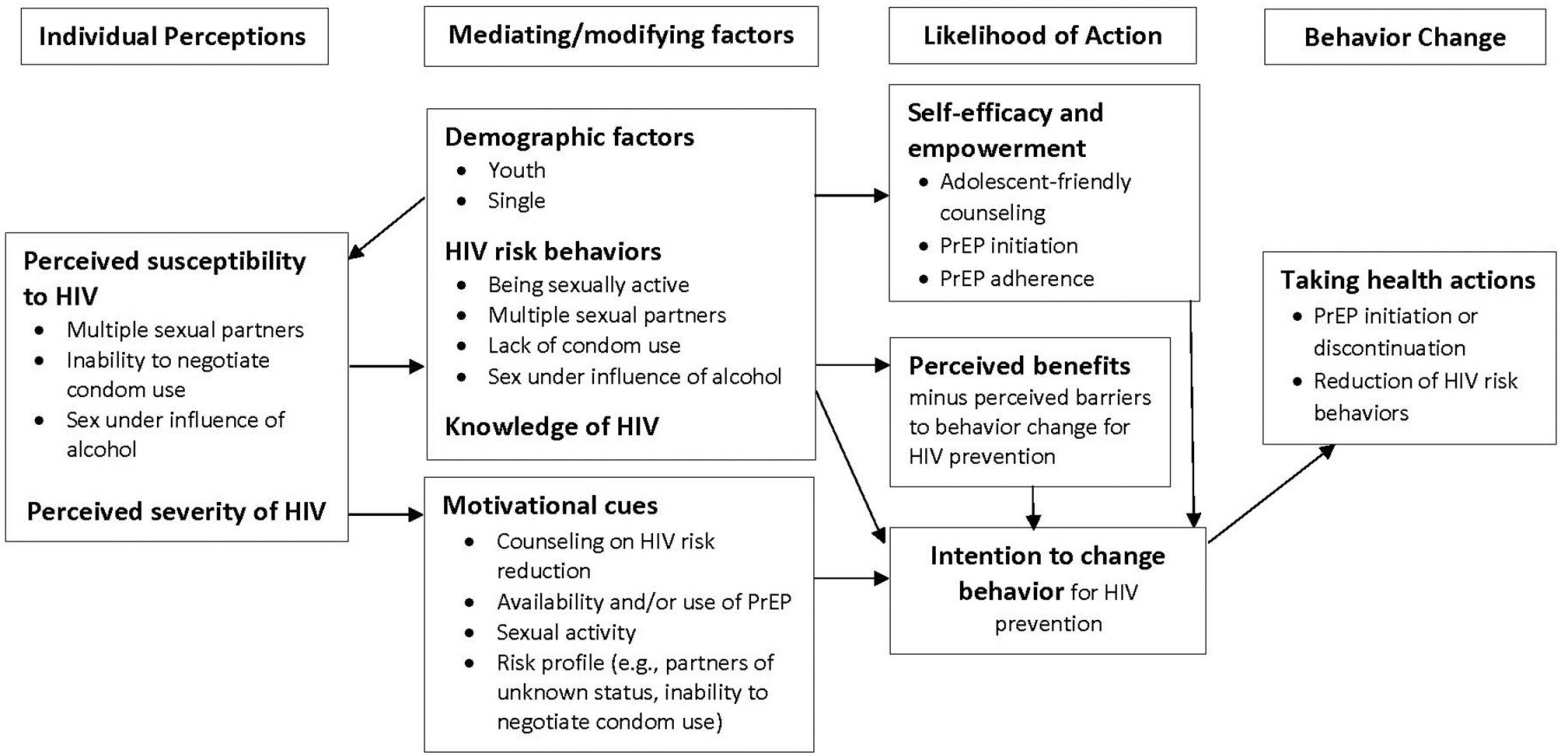
Application of the Expanded Health Belief Model to understanding perceived HIV risk and sexual behaviors among young women enrolled in a PrEP trial in Kenya.

Through the use of serial qualitative interviews over 1 year of follow-up in a PrEP trial, we learned that the young women participants perceived themselves as being at high risk of HIV infection because of their own individual behaviors, as well as those of their sexual partners. Additionally, these women perceived HIV/AIDS as serious disease with many equating it to death. These perceptions were key motivators for most to initiate PrEP and are consistent with the Expanded Health Beliefs Model (Figure 1), which postulates that individuals who perceive themselves at risk of acquiring a serious disease will be more likely to engage in risk reducing behaviors (4–6). However, the young women's high-risk perception was not static. Rather, it evolved as life circumstances and sexual behaviors changed, often reflecting intentional decisions made by the young women that were influenced by the counseling and other experiences in the study. Circumstances that lowered the young women's risk included knowing their partners' HIV status and moving from a relationship with a high-risk partner to a lower risk, stable partner (e.g., getting married). This dynamic risk perception informed participants' adherence and persistence with PrEP, as well as their use of condoms. Participants were aligning their perceived risk with use of HIV prevention tools in a way that is consistent with the concept of prevention-effective adherence (33). The relationship among risk perception, PrEP use, and condom use was variable, yet rational.

Many young women reported reducing condom use because they felt protected by PrEP, while others reduced both PrEP and condom use because they were able to protect themselves through relationships with less risk (e.g., stable monogamous partners with known HIV negative status). Interestingly, condom use in studies of serodiscordant couples has been reported to remain stable after initiation of PrEP (34, 35), potentially indicating a desire to maintain multiple forms of protection. Indeed, one participant in this study reflected the same view. These perceptions are important for informing counseling that promotes self-efficacy to use the HIV prevention tools most appropriate to their own needs. Condom access, as well as the desire to use condoms and the ability to negotiate their use to prevent other sexually transmitted infections and unwanted pregnancies also plays an important role and should also be included in counseling messages (36), as should the potential for unrecognized risks or changes in risk.

Our study suggests that providing PrEP as part of a comprehensive prevention package was an important cue to action that enabled women to understand their risks better and supported them to make adjustments to their lives and reduce their risks, even for those who discontinued PrEP use. Many HIV prevention trials have reported lower HIV incidence than expected even with modest overall PrEP use, including the MPYA study which had an HIV incidence rate of 0.7 per 100 person-years and overall adherence of 27% (23, 37, 38). These additional benefits included regular HIV testing and counseling, provision of male and female condoms, young women friendly HIV prevention services, and possible rational PrEP use (i.e., use of PrEP during periods of risk). While the goal of achieving behavior change through such services has been elusive in many settings (39), the addition of PrEP as a known effective, female-controlled, empowering tool may make be a kind of game changer for some women. Further exploration of potential mechanisms for behavior change is warranted and may inform how best to position PrEP among other HIV prevention options.

Strengths of this study include use of serial interviews over the course of a full year that allowed us to explore the issues of HIV risk and sexual behavior in a prospective, in-depth way. Additionally, longitudinal data collection reduced possible recall bias. One potential limitation is the potential social desirability bias associated with face-to-face interviewers. It is also possible that those who declined enrollment in the MPYA trial could have had different risk perceptions. Lastly, our findings should be interpreted in the context of a narrowly defined population—young women considered at high risk for HIV acquisition who had elected to join a PrEP trial. Future work should therefore explore risk perceptions and risk behavior in more diverse populations.

In summary, our study revealed that high HIV risk perception among young Kenyan women was heavily influenced by dynamic sexual behaviors and relationships, which were key motivators for PrEP use. Confidence in PrEP efficacy led to low perception of HIV risk among women opting to use PrEP; this association could be leveraged by HIV prevention programs to motivate PrEP adherence. Targeting young women who are already aware of their risk for HIV acquisition could increase uptake during PrEP roll-out, while innovative strategies could be used to educate other high-risk women to appraise their risk. Counseling within PrEP programs should be cognizant of the dynamic nature of HIV risk and continually adapt the counseling messages to meet young women's needs. Finally, the ability of young women to reduce their risk behavior during their participation in the PrEP trial speaks to PrEP as an empowering tool with potential for multifaceted behavior change. This empowerment may explain some of the low HIV incidence seen in PrEP programs even in light of modest PrEP adherence and persistence. Future work should explore how best to support young women with a variety of HIV prevention tools and integrate flexibility in delivery that adapts to the change in HIV risk status over time.

## Data Availability Statement

The raw data supporting the conclusions of this article will be made available by the authors, without undue reservation.

## Ethics Statement

The studies involving human participants were reviewed and approved by Kenya Medical Research Institute Scientific and Ethical Research Unit (KEMRI-SERU) and the University of Washington Human Subjects Division. All participants provided written informed consent.

## Author Contributions

KN, JMB, NM, EB, and JEH: conceptualizing and designing the study. NT and VO: acquired the data. VO, NT, BB, and KN: analyzed the data. KN and JEH: wrote the paper. All authors reviewed and edited the manuscript and approved the final version.

## Conflict of Interest

The authors declare that the research was conducted in the absence of any commercial or financial relationships that could be construed as a potential conflict of interest.

## Publisher's Note

All claims expressed in this article are solely those of the authors and do not necessarily represent those of their affiliated organizations, or those of the publisher, the editors and the reviewers. Any product that may be evaluated in this article, or claim that may be made by its manufacturer, is not guaranteed or endorsed by the publisher.
