# Supplementary material for: Dynamic Perceived HIV Risk and Sexual Behaviors Among Young Women Enrolled in a PrEP Trial in Kenya: A Qualitative Study
Source: Front Reprod Health. 2021 Aug 12;3:637869. doi: 10.3389/frph.2021.637869 (PMC9580724; doi:10.3389/frph.2021.637869)
Supplement: Supplementary file 1 [file Data_Sheet_1.DOCX]

SUPPLEMENTAL MATERIALS

MPYA Qualitative interview guides

30th November 2016

Version 2.6

These interview guides present the questions planned for each interview. The questions are written in standard formatting; instructions to the interviewer are italicized and/or bracketed. Exact wording may vary interview-by-interview to be appropriately responsive to the content of the answers, and some questions may be skipped if not relevant to the individual. However, no additional concepts will be pursued.

**Primary interview (enrollment)**

**Topic 1: Wisepill**

(*In this section, we seek to understand how the participant anticipates using the Wisepill device. This includes logistics related to the use of the device, as well as social factors, such as stigma, disclosure etc.)*

Tell me about the Wisepill container you will be using for your pills. What does it do?

In what ways do you think Wisepill will be helpful?

What are your concerns about Wisepill?

What do you think will be easy about using Wisepill?

What are the challenges related to using the Wisepill device?

- Probe about challenges with using the device as well as with relationships

How do you think the WP device will affect your relationship with your sexual partner(s)?

**Topic 2: SMS reminders**

*(For participants assigned to SMS reminders—please check with data team to find out if participant is randomized to the SMS arm before interview)*

In what ways do you think the SMS reminders will be useful?

What are your concerns about the SMS reminders?

- Probe for concerns about disclosure, relationships, adherence

What do you think will be easy about the SMS reminders?

What are the challenges you’ve encountered with the SMS reminders?

**Topic 3: HIV risk perception**

*(In this section we want to understand how the participant perceives her risk of HIV acquisition, how she perceives her risk compared to others in her social network, and her understanding of HIV transmission)*

What does HIV mean to you?

- Probe about if the participant found out her friend was HIV+, what would that mean to her?

How would you describe your risks for HIV infection?

- Probe about why the participant thinks she is at risk

How well do you think you know your risks?

How does your risk compare to that of your friends or others in your community?

- Probes related to # of sexual partners, marital status

**Topic 4: PrEP**

(*In this section we want to understand how participants are taking and using PrEP, how it might influence relationships, schedules, habits and behaviors, the perception of the future and what might make taking PrEP difficult or easy*)

*(For all participants)*

Tell me about PrEP. What does it do?

*Probe for protection against HIV generally and for the participant specifically.*

Tell me why you want to take PrEP.

How do you plan on taking PrEP?

What does PrEP mean to you?

What are the benefits of PrEP? What are the challenges of PrEP?

What are your concerns about taking PrEP?

How do you think it will affect your relationship with your sexual partner(s)?

- Has taking PrEP changed your other sexual behaviors?
- Has PrEP changed how you and your partner decide to use condoms?
  - - Probe about how and why
- Are there ways your sexual partner makes taking PrEP easier or harder? What ways?

(*If the participant has not disclosed her PrEP taking to partner, skip the below question*)

- How does your main partner feel about you taking PrEP?

In addition to what we’ve already discussed, can you tell me if there are other factors that influenced your decision to take PrEP?

Tell me about your future. Do you think PrEP will change your future? Why or why not?

Is there anything else you would like to tell me about taking PrEP?

**5. Wrap-up**

(*This section is to answer any questions the participant might have about the study and to address anything else not already covered in the interview*)

We have talked about many things today. Before we finish, I wonder if you have any questions for me or if you have any additional comments about your experience in MPYA or about the discussion today?

**Primary interview (3 months)**

**Topic 1: Wisepill**

(*In this section, we seek to understand how the participant experiences using the Wisepill device. This includes logistics related to the use of the device, as well as social factors, such as stigma, disclosure etc.)*

You’ve been taking PrEP for 3 months now.

Please tell me what it has been like using the Wisepill container to take PrEP.

How was it learning and using Wisepill?

How has Wisepill helped you?

What concerns do you have about using Wisepill? What are some of the challenges of using it? Can you give me some examples? In what ways is ________ a challenge?

*Probe on each challenge mentioned (e.g., device failure, network connectivity, size, color, battery life, device lighting with signal transmission, convenience).*

Who else seen your Wisepill container?

- If so, probe for how people react when they see the Wisepill container?
- If not, why?

What improvements would you make to Wisepill? Why?

**Topic 2: SMS reminders**

*(For participants assigned to SMS reminders—please check with data team to find out if participant is randomized to the SMS arm before interview)*

*(For participants receiving SMS reminders)*

As part of this study, you have also been receiving daily SMS reminders.

Tell me about the last time you received an SMS reminder. What did you do immediately after receiving it?

- *Probe about the individual’s particular experience. What was she doing? Where was she?*
- *What do you like about the reminders? What do you dislike? Why?*
- *Probe about content as well as the act of receiving the messages.*
- *In what ways are the reminders useful?*

How do the reminders what you were expecting when you joined this study? In what ways have they been different from your expectation?

How do the reminders help you with swallowing PrEP?

- If so, how?
- *Probe for specific examples.*

What concerns do you have about using SMS to support taking PrEP? What are the difficulties you face in receiving SMS reminders?

- *Possible probes: timing, privacy (especially in the case of sharing the phone), social acceptance of the reminders, network connectivity, device failure (e.g., phone not working), inconsistent phone use (no battery charge, changes in phone number).*

Who else has seen the SMS reminders?

- If so, who saw them? Tell me what happened?

Is there anything else you would like to tell me about receiving the SMS reminders that we haven’t talked about?

**Topic 3: PrEP**

(*In this section we want to understand how participants are taking and using PrEP, how it might influence relationships, schedules, habits and behaviors, the perception of the future and what makes taking PrEP difficult or easy*)

*(All participants)*

I would now like to ask you some questions about PrEP.

How has it been taking PrEP? What have you liked? What have you disliked?

- How do you use PrEP daily? How does it work for you?
- How do you usually take your PrEP? When was the last time you swallowed a PrEP pill?
- Probe for details-- *Where were you? Were other people present?*

What helps you take PrEP? What makes it difficult?

Please tell me about the most recent time you did not take a dose of PrEP.

*Probe in depth for the detailed story of the missed dose.*

Tell me about your experience of taking PrEP

- Is taking PrEP what you thought it would be like? Why or why not?

What does PrEP mean to you now that you’ve been taking it for a while?

What are the benefits of PrEP? What are the challenges?

What concerns do you have concerns about taking PrEP?

How long do you think you need to take PrEP?

How long do you think you would like to take PrEP? What influences your decision?

- What are they? Are they different than they were at the start of the study?

Everyone has stress in their lives, how did taking PrEP affect the stress in your life?

- How has this changed over time?

How has taking PrEP affected your relationship with sexual partner(s)?

- Has taking PrEP changed your other sexual behaviors?
- Has PrEP changed how you and your partner decide to use condoms?
  - - Probe about how and why
- Are there ways your sexual partner makes taking PrEP easier or harder? What ways?

(*If the participant has not disclosed her PrEP taking to partner, skip the below question*)

- How does your main partner feel about you taking PrEP?

1. **PrEP and risk perception**

*(In this section, we want to understand the relationship between taking PrEP and perception of HIV risk, as well as changes in other behaviors that may be risky)*

How has taking PrEP changed your other sexual behaviors?

- *Probe on condom use, number of partners*

What do you think about HIV now? What does it mean to you? How has this changed since you enrolled in MPYA?

What does HIV mean to you?

- Probe about if the participant found out her friend was HIV+, what would that mean to her?

How would you describe your risks for HIV infection now? How well do you think you know your risks? How does your risk compare to that of your friends or others in your community?

- Probe based on # of sexual partners, marital status

How well do you think PrEP protects you against HIV?

- How often do you need to take your PrEP pill to be protected from HIV transmission?

Are there other things that influence your decision to take PrEP now? What are they? Are they different than they were at the start of the study?

Tell me about your future. Do you think PrEP will change your future? Why or why not? How?

What else you would like to tell me about taking PrEP?

**5. Wrap-up**

(*This section is to answer any questions the participant might have about the study and to address anything else not already covered in the interview*)

We have talked about many things today. Before we finish, I wonder if you have any questions for me or if you have any additional comments about your experience in MPYA or about the discussion today?

**Primary interview (12 months)**

*(Interviewer should read previous transcripts for the participant prior to interview to understand how she has experienced taking PrEP and using Wisepill)*

**Topic 1: Wisepill**

(*In this section, we seek to understand how the participant experiences using the Wisepill device. This includes logistics related to the use of the device, as well as social factors, such as stigma, disclosure etc.)*

You’ve been taking PrEP for about one year now.

Please tell me what it has been like using the Wisepill container to take PrEP.

How has the Wisepill helped you? What do you like about it?

What concerns do you have about using Wisepill? What are some of the challenges of using it? Can you give me some examples? In what ways is ________ a challenge?

*Probe on each challenge mentioned (e.g., device failure, network connectivity, size, color, battery life, device lighting with signal transmission, convenience).*

How have people reacted when they see the Wisepill container?

What do you like about the Wisepill device?

What challenges have you encountered using the Wisepill device?

- Probe about challenges with device and relationships

What improvements would you make to Wisepill? Why?

**Topic 2: SMS reminders**

*(For participants receiving SMS reminders- Questions will vary if the participant is receiving the reminders daily or triggered by missed doses. Interviewer to consult the data team prior to interview to find out about the specific participant)*

As part of this study, you have also been receiving SMS reminders.

What is it like to receive SMS reminders every day? What do you like? What do you dislike? Why?

What is it like to receive SMS reminders when you miss a dose? What do you like? What do you dislike? Why?

- *Probe on supportive feelings, potential for “getting caught”.*

Which one do you prefer? Why? How was it to change from daily SMS reminders to reminders only when you miss a dose?

How do you feel about the reminders now that you’ve been getting them for a year?

- *Probe on SMS fatigue.*

How do you like receiving SMS reminders? If so, for how long would you like to receive them? Why?

When you joined this study, what were your expectations for the study? In what ways have they been different from your expectations?

- Probe about what the participants expectations were at enrollement

What concerns do you have at this time about using SMS to support taking PrEP? What are the challenges you face in receiving SMS reminders?

- *Possible probes: timing, privacy (especially in the case of sharing the phone), social acceptance of the reminders, network connectivity, device failure (e.g., phone not working), inconsistent device use (no battery charge, changes in phone number).*

Who else has seen the SMS reminders?

If so, who saw them? Tell me what happened?

What else you would like to tell me about receiving the SMS reminders that we haven’t talked about?

**Topic 3: PrEP**

(*In this section we want to understand how participants are taking and using PrEP, how it might influence relationships, schedules, habits and behaviors, the perception of the future and what makes taking PrEP difficult or easy*)

*(All participants)*

I would now like to ask you some questions about PrEP.

Tell me how has it been taking PrEP? What have you liked? What have you disliked?

**Probes**

In what ways do you use PrEP?

How well do you think PrEP protects you against HIV?

What helps you take PrEP? What makes it difficult? How has that changed now that it’s been one year?

Is taking PrEP what you thought it would be like? Why or why not?

What does PrEP mean to you now that you’ve been taking it for a while?

What are the benefits of PrEP? What are the challenges?

Do you have concerns about taking PrEP?

How long do you think you need to take PrEP?

How long do you think you would like to take PrEP? What influences your answer?

Tell me about the time/s you stopped taking PrEP at any point in the study? For how long did you stop? Why? Did you re-start? Why?

Everyone has stress in their lives, how was taking PrEP affected the stress in your life?

How has taking PrEP affected your relationship with sexual partner(s)?

- Has taking PrEP changed your other sexual behaviors?
- Has PrEP changed how you and your partner decide to use condoms?
  - - Probe about how and why
- Are there ways your sexual partner makes taking PrEP easier or harder? What ways?

(*If the participant has not disclosed her PrEP taking to partner, skip the below question*)

- How does your main partner feel about you taking PrEP?

1. **PrEP and risk perception**

*(In this section, we want to understand the relationship between taking PrEP and perception of HIV risk, as well as changes in other behaviors that may be risky)*

Has taking PrEP changed your other sexual behaviors?

- *Probe on condom use, number of partners.*

How do you think about HIV now? What does it mean to you?

- Probe about if the participant found out her friend was HIV+, what would that mean to her?

What are your risks for HIV infection now? How well do you think you know your risks? How does your risk compare to that of your friends or others in your community?

What other things that influence your decision to take PrEP now?

Tell me about your future. Do you think PrEP will have an impact on that? Why or why not? How?

What else you would like to tell me about taking PrEP?

**5. Wrap-up**

We have talked about many things today. Before we finish, I wonder if you have any questions for me or if you have any additional comments about your experience in MPYA or about the discussion today?
